# Progress in the Application of Porous Tantalum Metal in Hip Joint Surgery

**DOI:** 10.1111/os.14255

**Published:** 2024-10-16

**Authors:** Kaiming Ma, Zhijie Ma, Liangliang Cheng, Dewei Zhao

**Affiliations:** ^1^ Orthopaedic of Department Affiliated ZhongShan Hospital of Dalian University Dalian China

**Keywords:** Arthroplasty, Hip Joint, Hip‐Preserving, Porous Tantalum, Treatment Strategy

## Abstract

Porous tantalum metal is a new orthopedic implant material made of tantalum metal that has been processed by porous treatment. This material has various advantages, including high hardness, good ductility, good biocompatibility, and strong bone integration ability. Porous tantalum metal has performed well in clinical application, demonstrating excellent medium‐ to long‐term curative effects. The use of implant products made of porous tantalum metal, such as porous tantalum rods, porous tantalum hip prostheses, and porous tantalum augments (MAs), is gradually increasing in the clinical application of hip surgery, and these products have achieved excellent therapeutic effects in the middle and late stages of various hip diseases. In recent years, the combined application of porous tantalum metal and three‐dimenional (3D) printing technology to create personalized 3D‐printed porous tantalum metal has led to new development directions for the treatment of complex hip joint surgical diseases. This review presents a summary of the application of porous tantalum metal in hip surgery in recent years, including clinical treatment effects and existing problems. In addition, the prospect of progress in this field is promoted.

## Graphical abstract

Application of porous tantalum materials in hip joint surgery.

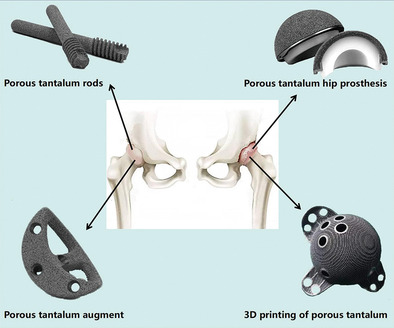

## Introduction

Hip joint disease is a common orthopedic disease that is mainly characterized by hip joint pain and functional limitations. If not treated in the early stage, this disease will further aggravate the symptoms of patients, seriously affect their physical and mental health and social function, and eventually cause substantial psychological and economic burdens.^1^ To date, the diagnosis and treatment of hip joint surgical diseases are closely related to implants, and the related characteristics of implants greatly impact the surgical effect. Although autologous or allogeneic bone grafts have the advantages of closely resembling the original bone structure and causing less rejection, the implanted bone may collapse due to inadequate nutritional supply, resulting in loss of function.^2^ Previous implant materials, such as titanium alloys, have achieved great success in the treatment of hip joint diseases, but many defects are present.^3^ For example, a high elastic modulus, low shear strength, corrosiveness, and immune reactions may lead to implantation failure.^4^

**Figure 1:**
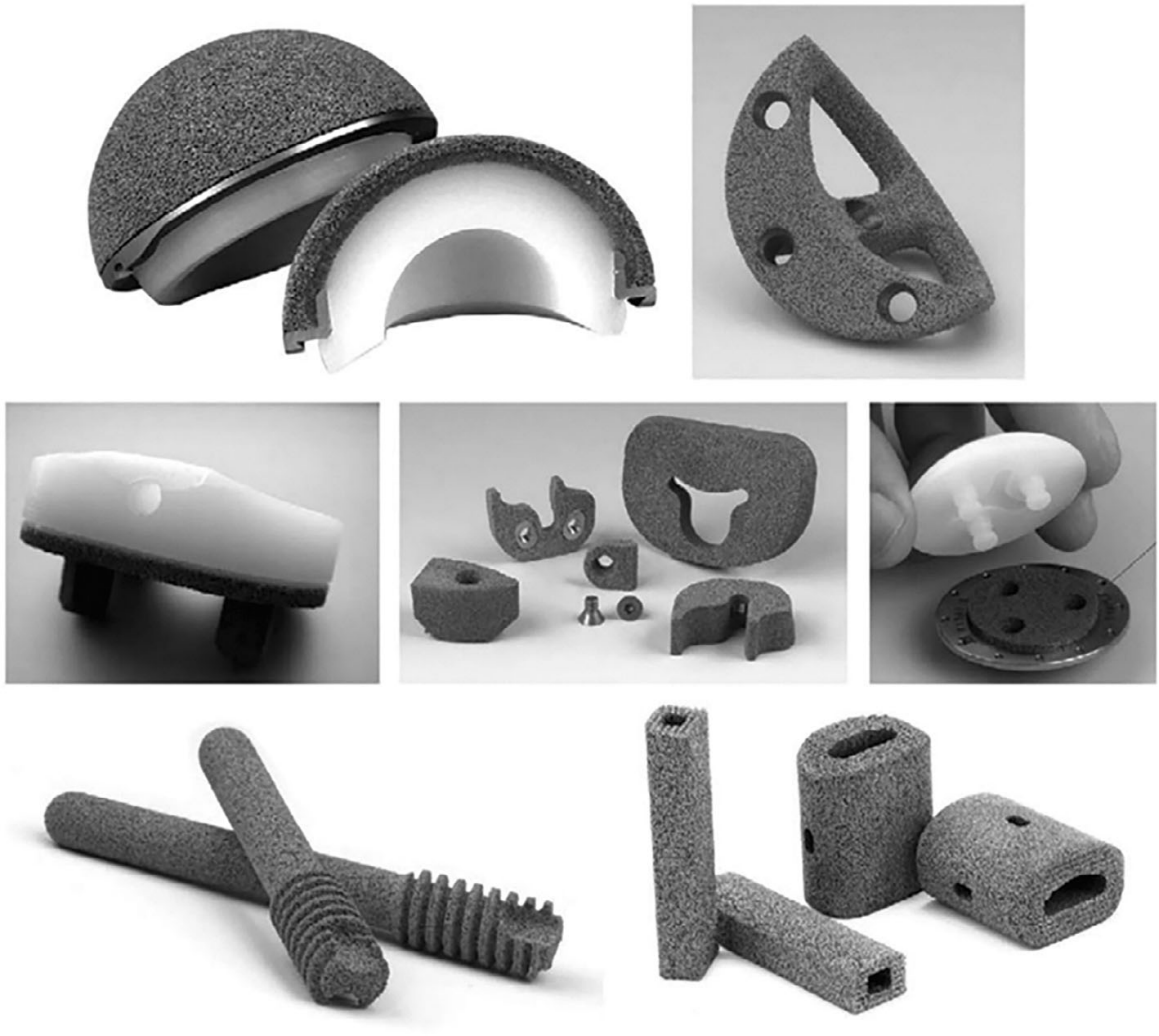
Multiple orthopedic applications for porous tantalum. Top row: monoblock acetabulum, and a revision acetabular augment. Middle row: monoblock tibia, revision TKA augments, and salvage patella button. Bottom row: osteonecrosis implant and spine arthrodesis implants (Courtesy of Zimmer, Warsaw, IN).^8^

Tantalum is a rare metal that is hard and ductile; as an implant material for the human body, tantalum metal does not react with body fluids and has no stimulating effect on human tissue cells.^5^ Tantalum metal was first used in orthopedics in 1940 and has since been widely used in the medical industry.^6^ However, the high elastic modulus, high density, and high cost of pure tantalum hinder its application in orthopedics.^7^ Until Zimmer, Inc. successfully produced commercial trabecular porous tantalum metal, solving the problem that originally limited the application of pure tantalum (Figure 1), and widely used in clinical, especially in the application of various hip joint diseases achieved good clinical follow‐up effect.

With the development of technology, especially three‐dimensional (3D) printing technology in recent years, great progress has been made in the application of porous tantalum in hip surgery. This paper presents a review of related research reports from the past 5 years, a summary of the clinical application effect and existing problems of porous tantalum implants in hip joint surgery, and a proposal of the prospect of development.

## Methods

The search procedure is as follows. Source: The first author conducted a literature search in January 2024 using the keywords “hip” and “tantalum.” A literature search was conducted from 2019 to 2024 using PubMed, Web of Science, and Chinese National Knowledge Infrastructure (CNKI), and the types of literature searched were original research articles, reviews, commentaries, case reports, meta‐analyses, etc. The amount of retrieved literature was 442 articles.

Inclusion criteria: (i) Articles related to hip joint surgery and porous tantalum; (ii) Articles on the physicochemical properties and biological properties of porous tantalum metal; (iii) Research with sufficient evidence and solid arguments. Exclusion criteria: (i) Articles with duplicated content; (ii) Articles that do not correspond to or are not relevant to the topic described; (iii) Articles with a long history.

**Figure 2:**
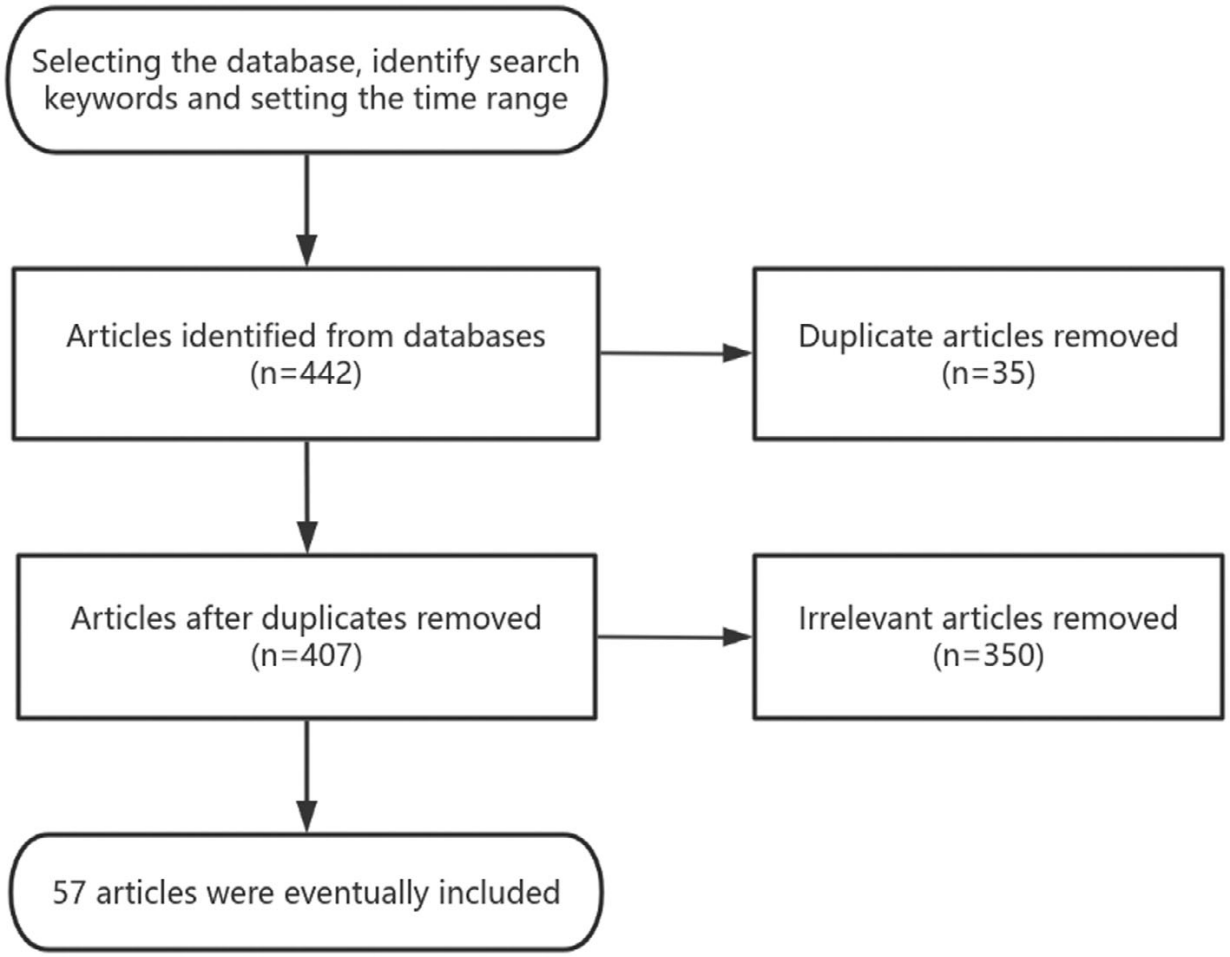
Literature screening flowchart.

Quality assessment: a total of 442 relevant articles were initially obtained, and the collectors assessed the validity and applicability of the included articles by reading the titles and abstracts of the articles for preliminary screening; repetitive studies and irrelevant articles were excluded, and a total of 57 articles in Chinese and English were finally included in the review, which was obtained from the databases of PubMed, Web of Science, and CNKI. A brief flow chart is presented in Figure 2.

## Advantages of Porous Tantalum Product Implant Materials

As a new orthopedic implant material, porous tantalum metal is a finished product made of tantalum metal that has undergone porous treatment. In terms of mechanical strength, porous tantalum retains the good hardness and ductility of tantalum metal, which can provide sufficient mechanical support for new bone tissue.^9^ In terms of structure, the surface of porous tantalum material is rich in honeycomb gaps, the porous tantalum prosthesis used for implantation materials generally contains many microporous structures with diameters of 400–600 μm and porosities of approximately 75%–85%, and the internal voids are interconnected. This structure increases the contact area between the prosthesis and bone while reducing the density and elastic modulus of the tantalum metal.^10^ Compared with other materials, such as titanium alloy, the special elastic modulus of porous tantalum metal is more similar to that of cortical bone, which helps to transfer the load and effectively reduce the shielding effect (Table 1).^11^ This metal is beneficial for maintaining bone density around the implant and reducing long‐term bone loss around the implant. In addition, the porous structure gives the material a rough surface, providing sufficient initial stability after implantation in the host bone.^12^ Therefore, the excellent physical and chemical properties of porous tantalum can provide good stability for patients at the initial stage of material implantation and create enhanced conditions for bone growth.

**Table 1:** Performance comparison of tantalum with other bone implant materials.

	316L stainless steel	Cobalt‐based alloy	Titanium	Titanium alloy	Porous tantalum
Biocompatibility	○	◎	•	•	•
Modulus of elasticity	○	○	◎	◎	•
Mechanical strength	•	•	◎	•	◎
Biological activity	○	○	○	○	◎
Corrosion resistance	○	◎	•	•	•

*Note*: •‐excellent, ◎‐good, ○‐poor.

In addition to having suitable physical properties and corrosion resistance, the ideal implant material should not be toxic to the human body and should have good biocompatibility, osteogenic induction ability, and bone‐like biomechanical properties.^13^ To date, porous tantalum has shown good biocompatibility, and *in vivo* and *in vitro* experiments have shown satisfactory results without obvious inflammation or allergic reactions. Studies have shown that all types of cells can survive on the surface of porous tantalum and maintain normal cell activity and growth. Bruggemann *et al*.^14^ collected blood samples from patients receiving porous tantalum implants and analyzed the blood tantalum concentration and the total number of white blood cells and lymphocyte subsets. The results showed that the blood tantalum concentration increased with an increasing number of operations involving porous tantalum implants, but the overall concentration was low (Two outliers presented with a maximum value of 0.44 mg/L). There is no safe blood concentration standard for tantalum. However, compared to other similar metals, such as chromium and cobalt, serum levels of >17 mg/L for chromium >19 mg/L for cobalt are associated with metallosis. The concentration of tantalum in this result is notably low and does not cause clinical symptoms or immune reactions such as lymphocyte elevation. Jiang *et al*.^15^ Conducted cytotoxicity tests on porous tantalum *in vitro* and micronucleus tests on mammalian bone marrow cells *in vivo*, and the results showed that porous tantalum had no cytotoxicity or genotoxicity.

What's more, porous tantalum can promote bone growth and integration. Studies have shown that the implantation of porous tantalum *in vivo* can upregulate the expression of osteogenic genes at various stages.^16^ Moreover, due to its extensive 3D internal space and high pore size and porosity, porous tantalum can promote the growth of bone and soft tissue into a pore, resulting in new bone formation and bone deposition around the implant, leading to bone integration.^17^ Long‐term biological stability increases after secondary bone reconstruction, which is the most important factor in achieving long‐term implant stability.^18^ Studies have shown that the appropriate pore size of a scaffold determines the extent of bone tissue repair. When the pore size is <50 μm, only fibrous tissue and noncalcified bone‐like tissue can form inside the pore. When the pore size is 100–200 μm, osteoblasts can proliferate and migrate into the pore. Finally, when the pore size is >300 μm, vascularized bone tissue can form. This material is conducive to bone regeneration; thus, the pore size of porous tantalum (400–600 μm) can contribute to the promotion of bone growth.^19^ In addition, appropriate porosity and pore structure containing both small and large pore sizes can further facilitate nutrient transport and allow cell penetration into the scaffold to form new tissue. Wang *et al*.^20^ fabricated porous tantalum scaffolds with six combinations of pore structures. The osteogenesis, angiogenesis, and biosafety of different porous tantalum scaffolds were evaluated by *in vitro* cell experiments and *in vivo* animal experiments. The results showed that the trabecular porous tantalum scaffolds with a pore size of 500 μm, a pore distribution range of 200–1200 μm, and a porosity of 70% showed optimal bone repair potential *in vitro* and *in vivo*. Furthermore, porous tantalum can promote muscle generation and differentiation, which is conducive to inward growth and adhesion of muscle.^21^

## Clinical Application of Porous Tantalum Products

### Porous Tantalum Rods

For younger patients who have femoral head necrosis but have not yet collapsed, the main research direction to date is to slow the progression of femoral head necrosis through conservative treatment and delay the timing of hip replacement surgery. Core decompression is a commonly used and effective method that can effectively reduce the pressure in the femoral head, relieve pain, contribute to the formation of revascularization in the necrotic area, and promote the formation of new bone.^22^ However, drilling results in a lack of subchondral bone surface, resulting in inadequate mechanical support for the femoral head. This increases the risks of femoral head collapse and subtrochanteric fracture.^12^ Therefore, core decompression combined with bone grafting with vascular pedicles is often used for clinical treatment. Bone flaps can promote the reconstruction of blood circulation and provide mechanical support to maintain mechanical stability.^23^ However, the disadvantages of this method are complicated surgery, a large amount of blood loss, and donor area damage; thus, its application is limited.

**Figure 3:**
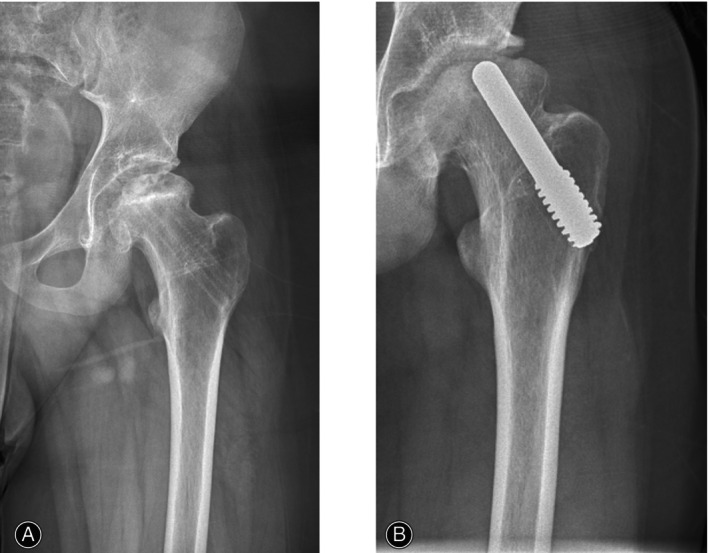
Preoperative radiograph prior to tantalum rod insertion (A) and postoperative radiograph at 14 months with tantalum rod *in situ* (B).^27^

Porous tantalum rod implantation is a new method for treating early necrosis of the femoral head that has emerged in recent years. This method involves the replacement of the bone flap with a porous tantalum rod to fill the residual defect area after decompression of the pulp core, and it promotes the reconstruction of blood circulation in the avascular necrosis area of the femoral head.^24^ This kind of surgery has been widely used because of its advantages, such as simple operation, short exposure time, high safety, and few complications. Numerous studies have shown that porous tantalum rods can achieve satisfactory effects on femoral head necrosis, promoting the recovery of hip joint function after surgery and alleviating pain levels.^25^ Li *et al*.^26^ followed 45 patients with femoral head necrosis who underwent pulp core decompression and porous tantalum rod implantation combined with autologous bone transplantation in Ficat stage II, and the results showed that the postoperative hip recovery, Harris score, visual analog score (VAS), and complication rate were better in the Ficat stage II group than in the control group without a tantalum rod. Hussaini *et al*.^27^ also applied tantalum rods for the treatment of femoral head necrosis in children. Children of age group 9–18 years with femoral head necrosis received porous tantalum rod treatment. After an average follow‐up of 18.2 months, the rate of femoral head collapse did not significantly increase compared with that before surgery, and the symptoms significantly improved (Figure 3A,B). The ability of tantalum rod implantation to prevent necrosis and collapse of the femoral head in children was demonstrated. However, the extensive application of tantalum rod implantation therapy in the field of pediatrics still requires considerable supporting evidence; moreover, as the femoral heads of children are in the growing stage, whether tantalum rods can be adapted over a long period still needs to be determined in new cases, and long‐term follow‐up studies are needed.

Although tantalum rod implantation has been described as a treatment for slowing the progression of necrosis and the collapse of the femoral head, there are significant differences in the therapeutic effects of tantalum rods in different studies.^28^ As the number of applications increases, failures are being reported. For this reason, many scholars have carried out analyses of tantalum rod treatment failure cases, hoping to determine the factors related to treatment failure. Su *et al*.^29^ followed up on the postoperative collapse of the femoral head in 77 patients who received porous tantalum rod implantation and reported that hormonal necrosis of the femoral head, the extent of the necrotic area, and the location of the necrotic area in the weight‐bearing area were independent factors affecting the collapse of the femoral head after porous tantalum rod implantation for early nontraumatic necrosis of the femoral head. Liu *et al*.^30^ propose that abnormal bone reconstruction around porous tantalum rods is an important factor causing the early failure. The trabeculae volume and morphology after the abnormal bone reconstruction were poorer than those of the normal femoral head. Which may be related to biomechanical stress distribution and chronic inflammation infiltration. Studies have shown that other factors, such as disease stage, the use of corticosteroids, and the volume and location of necrotic lesions, can influence the therapeutic efficacy of porous tantalum rods.^8^

For patients with failed tantalum rod implantation, continued conservative treatment has lost its value, and this issue can only be addressed with hip replacement at this time. However, due to its presence in the affected hip, the additional step of removing the tantalum rod during surgery may lead to increased blood loss, bone loss, prolonged operation time, and potential risk of trochanteric fracture.^31^ Moreover, removing the tantalum rod will generate substantial tantalum debris at the original implant site.^32^ To solve this problem, Zhao *et al*.^33^ conducted a study on the angle of tantalum rod removal direction, comparing patients who adopted an anterograde approach (moving the tantalum rod from the tip to the counterpart of the lateral femoral cortex) or a retrograde approach (moving the tantalum rod from the counterpart of the lateral femoral cortex to the proximal end). The results showed that the operation time, blood loss, amount of tantalum fragments, and radiation transmission of the retrograde approach were greater than those of the anterograde approach, indicating that anterograde surgery is a simpler and more effective method that provides value for tantalum rod removal. In addition, Cho *et al*.^34^ chose to retain tantalum rods and perform hip joint surface replacement for treatment. All postoperative scores of the 10 patients who underwent this surgical method were better than those before surgery, and no complications occurred. In this study, tantalum rods did not need to be removed, and the presence of tantalum rods could still play a supporting role and reduce the risk of femoral neck fracture after hip joint surface replacement, making it a valuable reference treatment. However, the author reported some cases of tantalum debris caused by contact with tantalum rods during surgery. These issues need to be resolved in the future.

In recent years, on the basis of simple porous tantalum metal rod implantation, many new methods have been developed and improved upon by scholars, and further positive clinical effects have been achieved. Huang *et al*.^22^ prepared a new type of porous tantalum material by using slip‐casting powder through filling technology; the physical properties of this material are similar to those of porous tantalum rods produced by Zimmer Trabecular Metal Technology, which is the most commonly used material in the market. After the clinical treatment of 21 patients, the efficacy of the new type of porous tantalum material was found to be similar to that of the Zimmer tantalum rod while effectively reducing cost. Mesenchymal stem cells cultured on tantalum rods have also been proposed to promote bone regeneration.^28^ However, the value and clinical application possibilities of these new technologies need additional, comprehensive research and reporting.

Although the porous tantalum rod has achieved good results in the early treatment of femoral head necrosis, its application has some limitations. For example, the porous tantalum rod can only serve as a single‐point support. It is currently accepted that porous tantalum rod implantation is only suitable for patients with small necrotic areas on their femoral heads, and it is challenging to provide sufficient support for patients with multiple lesions and large necrotic areas. This difficulty results in poor treatment.^25^ Therefore, future research on the risk factors for the use of porous tantalum rods and the innovation of technologies to improve their therapeutic effect remain necessary.

### Porous Tantalum Hip Prosthesis

Total hip arthroplasty (THA) is the most important treatment for end‐stage hip disease that can immediately solve the problems of hip pain and dysfunction.^35^ The metal materials used for artificial total hip replacement have continuously changed with the development of technology. Porous tantalum hip prostheses are metal materials with a high friction coefficient that can be closely combined with the host bone in the early stage of implantation to obtain good initial stability. Moreover, the porous structure provides good bone growth into the pore and integrates with the host bone to achieve biological fixation of the prosthesis. Finally, the long‐term stability of the prosthesis is achieved. Therefore, this topic is receiving increasing attention from clinicians. A study that included 398 cases of porous tantalum femoral prostheses showed that porous tantalum could significantly enhance the stability of the prosthesis.^36^

Rambani *et al*.^37^ conducted a direct comparison between tantalum acetabular cups used in primary total hip replacement and traditional titanium–acetabular cups in patients and observed that tantalum prostheses were superior to titanium prostheses in terms of postoperative prosthesis survival, bone integration performance, and reduction in osteolysis and mechanical loosening. Porous tantalum metal could be used on the acetabular side to obtain good clinical results, and its structure contributed to its application in femur‐side prostheses. Motomura *et al*.^38^ compared the efficacy of a prosthesis coated with a porous tantalum surface at the proximal end of the femur stem and a traditional rod prosthesis coated with a titanium fiber network surface and reported that the porous tantalum femur stem bone had enhanced penetration and could provide early, strong fixation. In this study, prostheses with proximal porous structures were used. Due to the significant difference in hardness between the epiphyseal layer of the femoral shaft and the femoral stem prosthesis, bone growth near the proximal femoral stem was limited, which increased the risk of fracture at this location and increased the difficulty of revision surgery in the long term. To solve this problem, the application of porous metal to the proximal end of the femoral stalk to promote bone growth has been attempted, and some experimental results have supported this approach. However, some studies have reported opposing findings, indicating that traditional fully coated porous titanium prostheses are slightly superior to proximal‐coated porous titanium prostheses in promoting bone growth.^39^ Therefore, in future research on porous tantalum femoral stem prostheses, attention should be given to comparisons with fully coated porous tantalum prostheses.

**Figure 4:**
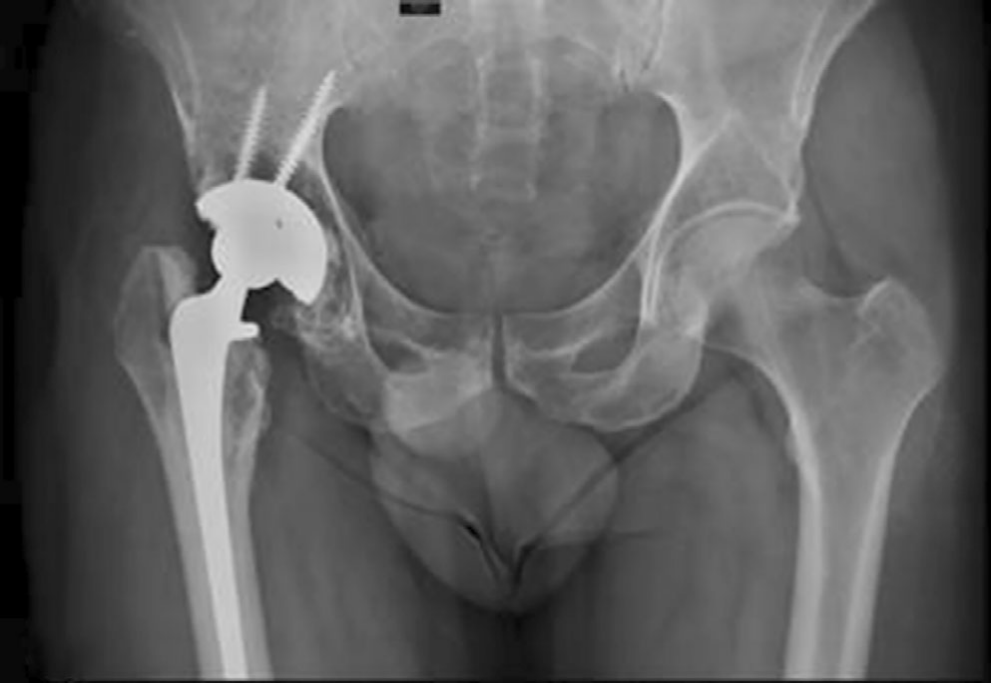
Pelvic X‐ray film performed 96 months postoperatively shows a revision shell cup fixed with screws.^43^

Howie *et al*.^40^ conducted a 2‐year follow‐up of 66 patients who had received a tantalum acetabular prosthesis without auxiliary screws or a titanium acetabular prosthesis with auxiliary screws and reported that there were no significant differences in implant displacement, symptoms, or functional scores between the two groups. This result proved that the good fixation ability of porous tantalum acetabular prostheses can reduce or prevent screw implantation and reduce operative time and intraoperative bleeding. The interconnected microporous structure, similar to the trabecular bone structure, helps additional new bone grow into the implant and improves the long‐term stability,^41^ which is generally considered the main reason for the good fixation of porous tantalum acetabular prostheses. In addition, porous tantalum is widely used in artificial total hip replacement surgery under other complex conditions. For example, in patients with malignant tumors requiring pelvic irradiation, a high rate of replacement failure has been reported in previous studies when a total hip replacement is needed. Early implant loosening and failure rates can be as high as 44%–52% at mid‐term follow‐up in traditional THA with both cemented and uncemented components.^42^ The reason for this phenomenon may be that osteoblasts are more sensitive to radiation than osteoclasts are, and a decrease in the number of osteoblasts affects the mineralization of the matrix. To address this challenge, Paolis *et al*.^43^ chose to follow up with 12 patients who had undergone hip replacement with a porous tantalum prosthesis and received an average radiation dose of 5500 cGy; the results showed that none of the patients had to undergo revision surgery due to aseptic loosening (Figure 4). However, to date, there is no theory to explain why porous tantalum prostheses have achieved excellent performance in patients with malignant tumors, and there are few relevant reports on this topic, which requires further supportive data and in‐depth research in the future.

In summary, porous tantalum hip prostheses have become an ideal material for hip replacement due to their excellent properties, which have been clinically validated, and good results have been achieved.

### Porous Tantalum Augment

Acetabular defects are complicated conditions in the treatment of hip joint surgical diseases, especially for patients for whom hip joint replacement requires revision. The removal of the original replacement prosthesis inevitably leads to the occurrence of acetabular bone defects, and acetabular reconstruction technology is required to fill the original defect. Traditional reconstruction techniques include giant cups, bone cement shells with allografts, rings or cages, and structural allografts. However, these methods have limitations, such as insufficient initial stability, risk of graft absorption, and easy long‐term mechanical failure.^44^

**Figure 5:**
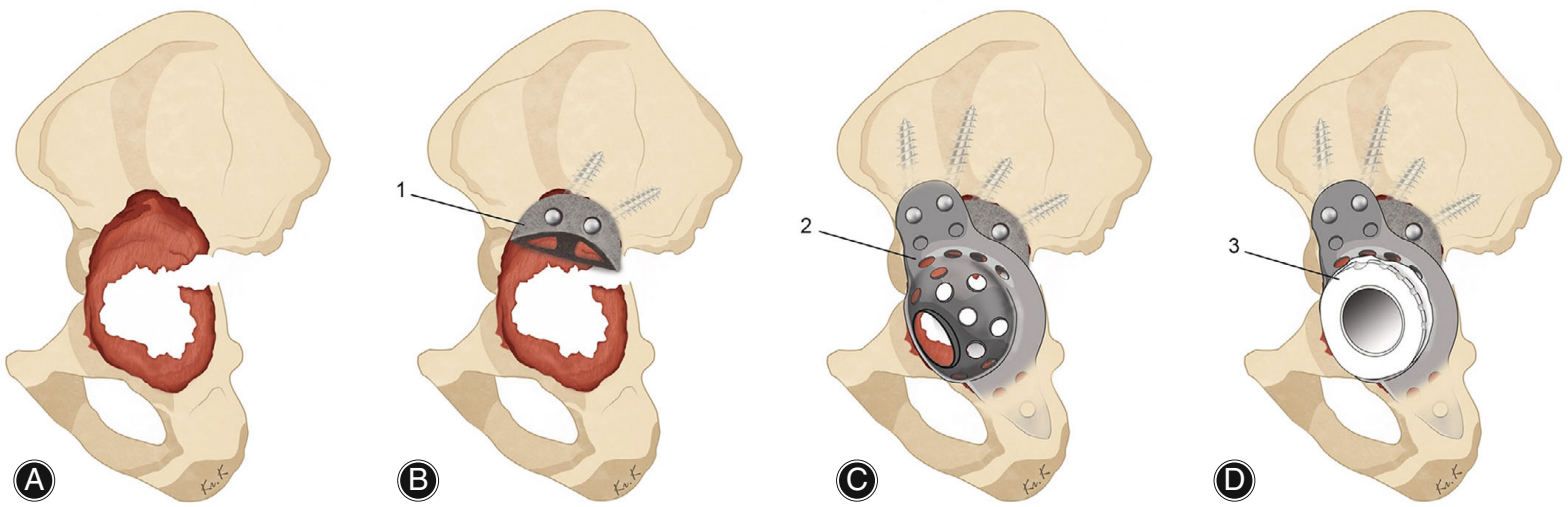
Illustration of revision hip arthroplasty with acetabular reinforcement ring (ARR) combined with an augment (MA). Paprosky type IIIB acetabular defects (A), MA fixation and bone grafting (B), cage fixation (C), and cementing polyethylene liner (D). (1: metal augment [MA], 2: acetabular reinforcement ring [ARR], 3: polyethylene liner).^48^

Studies have shown that porous tantalum may represent a promising option to address acetabular bone defects.^45^ Porous tantalum augments, as a representative, can be used as void fillers of different shapes to help reconstruct acetabular bone defects. With their excellent inherent properties, porous tantalum augments have been widely used and achieved good mid‐term and long‐term results.^46^ Alqwbani *et al*.^47^ followed 48 patients who underwent acetabular revision using porous tantalum acetabular prosthesis and augmentation technology for an average of 6.25 years. All the interfaces of the hips remained well‐fixed, and no patients required a second revision operation. Pain scores, functional scores, and patient satisfaction scores all showed significant increases. In this report, the porous tantalum acetabular prosthesis combined with the augmentation technique achieved excellent results in many affected hips with Paprosky type III bone defects in the selected patients, demonstrating the therapeutic effect of this approach in the treatment of severe bone defects. However, no patients in the selected patients had pelvic discontinuity, and when the acetabular defect was large or combined with pelvic discontinuity, traditional porous tantalum augmentation alone could not effectively fix the pelvis, and the treatment faced great challenges. In this case, the use of porous tantalum augmentation combined with other acetabular reconstruction techniques to bridge the pelvis could be increasingly advantageous. The acetabular reinforcement ring (ARR) is a type of prosthesis that could be used to connect and reinforce the pelvis with large bone defects. Due to the lack of filling of bone defects, the ARR is often used in combination with allograft bone. However, when the graft bone is absorbed or cannot be fused with autogenous bone, the ARR loses its fixation and fails. Porous tantalum augments can be used as an alternative to allograft bone grafts to provide adequate support for the ARR. Kim *et al*.^48^ followed up on 10 Paprosky type III patients who underwent THA revision with ARRs combined with porous tantalum augments for more than 8 years. Except for one patient who developed a prosthetic joint infection, the prostheses of the affected hips were all well‐fixed, and their symptoms significantly improved (Figure 5A–D). It has been proven in clinical follow‐ups that the use of an ARR combined with porous tantalum augmentation can yield satisfactory medium‐to‐long‐term results. In addition to porous tantalum augments combined with ARRs, the combined application of augments and other acetabular reconstruction techniques has been extensively studied and achieved good results.^49^

Although porous tantalum augments have good clinical results, they still have some shortcomings and room for improvement. Traditional augments are mass‐produced according to standard size and shape. While an appropriate prosthesis size can be selected according to different patients, in most cases, the residual bone mass of acetabular defects needs to be articulated. Moreover, the defect shape of each patient may still not be perfectly matched after articulation, resulting in a gap between the prosthesis and the host bone after implantation, which ultimately affects the lifespan of the prosthesis. Tantalum implants prepared by this traditional method have certain disadvantages, such as irregular apertures, unsatisfactory mechanical properties, unpersonalized customization, and high manufacturing costs,^50^ especially for patients with complex conditions, severe bone defects, or pelvic discontinuity, who often fail to receive good results.^51^ In addition, porous tantalum augments often require a combination of bone cement and acetabular cups.^9^ Long‐term loosening of the binding interface may occur, and these factors will eventually affect the service life of the prosthesis and limit its wide application.

### 3D Printing of Porous Tantalum

**Figure 6:**
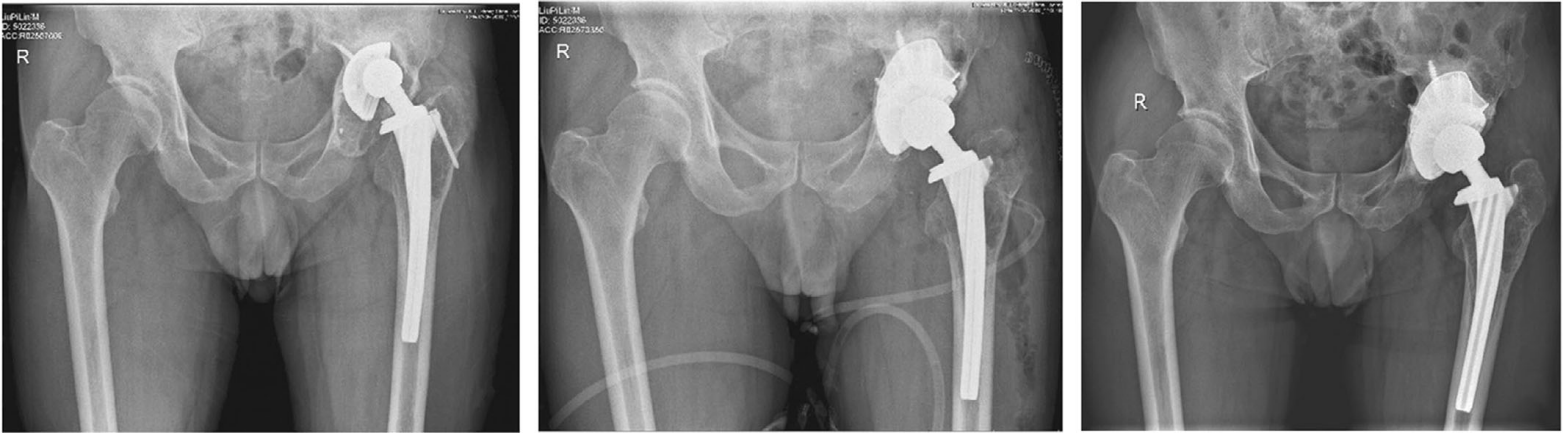
Radiographs showing an example of the use of a 3D printed tantalum augment (left hip) for the reconstruction of a Paprosky type IIB defect: (A) preoperative, (B) postoperative, and (C) last follow‐up anteroposterior (AP) radiographs at 3 years.^53^

In recent years, 3D printing technology has gradually been applied in the field of orthopedics, and its emergence has solved the abovementioned problems to a certain extent, especially in the treatment of acetabular bone defects. With 3D printing technology, personalized prostheses can be created according to the imaging data of different patients, which match the bone defect well in terms of geometric shape and completely fit the host bone, completely solving the matching problem between the prosthesis and the bone anatomy of the patient to obtain initial stability. The navigation template achieved by 3D printing technology can restore the anatomical structure of the acetabulum so that the surgeon can intuitively understand the 3D shape of the acetabulum bone defect to easily and accurately formulate an individualized surgical treatment plan, which can significantly reduce the operation time and control postoperative complications.^52^ In terms of material selection, titanium is still the most commonly used 3D printing material, but previous studies have shown that porous tantalum has a better ability than titanium to promote cell adhesion, proliferation, and biomechanics.^6^ Fan *et al*.^17^ compared the biomechanical properties of 3D‐printed porous tantalum and porous titanium scaffolds and concluded that porous tantalum scaffolds were superior to titanium scaffolds in terms of compressive resistance and deformation uniformity, and the stress–strain parameters of tantalum scaffolds were close to those of the host bone. Ying *et al*.^53^ used 3D‐printed porous tantalum augments to treat seven cases of acetabular bone defects. After a follow‐up period of 2.8–4.3 years, clinical symptoms were determined to be significantly reduced in all patients, and no mechanical loosening or complications were observed between the augments and the acetabulum (Figure 6A–C).

To date, 3D‐printed porous tantalum still faces many challenges in the treatment of acetabular bone defects. On the one hand, due to the difficulty of 3D printing technology in clinical application, it is necessary to determine the effective bone mass in the reconstruction process, overcome the influence of metal artifacts, and ensure the accuracy of the model, which requires the experience of doctors and communication and cooperation with engineers.^54^ On the other hand, due to technical and economic reasons, the amount of tantalum metal used in 3D printing applications and in research is far less than that available for titanium alloys and other materials, which brings great challenges to its application and implementation. Moreover, there has been clinical research on 3D‐printed porous tantalum for more than 10 years, although it has shown good early stability and bone growth ability in small sample clinical studies. However, the lack of mid‐to‐long‐term follow‐up periods and control studies makes its application unconvincing.

In addition, the immaturity of the technology is currently a primary challenge of its development. 3D printing technology can change the physical and chemical properties of materials by adjusting various processing parameters, such as the laser energy, scanning gap, scanning speed, and layer thickness, but the optimization of one physical or chemical property through this method is often accompanied by changes in other physical and chemical properties.^55^ The balance of various physical and chemical properties to maximize the advantages of finished porous tantalum needs further optimization and adjustment.^56^ Moreover, changes in the production method and physical and chemical properties can impact the biological properties of the material, which must be studied in relevant experiments in the future; on this basis, continuous coordination and improvement in production technology can be performed.^50^ Third, the application of surface modification technology to 3D‐printed porous tantalum is a future research direction. This method involves the application of a biomaterial coating and surface treatment to the surface of 3D‐printed porous tantalum to improve its bone conductivity, bone inductance, and antibacterial properties. Although this technology has been applied to porous tantalum metals to achieve different results in research, there have been no clinical reports or experimental studies performed on 3D‐printed porous tantalum, and whether its performance can be improved after surface modification needs to be verified through further experimentation and clinical observation.

## Summary and Outlook

Porous tantalum is a type of artificial implant material with excellent performance. This material has an appropriate elastic modulus, excellent mechanical strength, good corrosion resistance, good biocompatibility, and good bone integration ability due to its unique physical and chemical properties. In recent years, porous tantalum has increasingly been applied in orthopedic clinical treatment, especially in hip joints. The effects of porous tantalum rods, porous tantalum hip prostheses, porous tantalum augments, and other products have been fully verified, and these materials have been shown to have excellent medium‐ and long‐term therapeutic effects. However, the disadvantage of standardized prostheses is that they cannot perfectly adapt to the needs of every patient. With the continuous progress of technology, standard tantalum metal materials can no longer meet the increasing clinical requirements for surgical results, and the high price of standard tantalum metal materials limits their widespread promotion and application in the market.^57^ With the development of 3D printing technology in recent years, customized porous tantalum prostheses may be able to fill this gap and become the primary direction for the future clinical development of porous tantalum. With the advantages of personalized 3D‐printed porous tantalum, several clinical studies have reported that its clinical effect is better than that of other implants, but this new technology is still limited by its short follow‐up time, lack of control studies, immature technology, and other problems, which requires further relevant experimental studies and improvements in production technology.

## Author Contributions

Conception and design of the study: Dewei Zhao. Collection of the references, mapping of figures, and writing most of the manuscript: Kaiming Ma. Writing of the manuscript writing and revision process: Zhijie Ma. Checking the manuscript: Liangliang Cheng and Dewei Zhao. All authors listed have made a substantial contribution to the work.

## Conflict of Interest Statement

The authors have no financial or proprietary interests in any material discussed in this article.

## Ethics Statement

This review does not require ethical clearance from any institutional level committees, as any data or information being collected have already been made available for use through published peer‐review literature. No other identifying information or data will be used in this study. Outcomes of the study will be disseminated as journal manuscripts and conference presentations.
